# The complete chloroplast genome sequence of *Cerasus jingningensis* (Rosaceae), an endemic plant to Zhejiang province, China

**DOI:** 10.1080/23802359.2019.1676179

**Published:** 2019-10-11

**Authors:** Ming Jiang, Junfeng Wang

**Affiliations:** a Zhejiang Provincial Key Laboratory of Plant Evolutionary and Conservation, College of Life Science, Taizhou University, Jiaojiang, China;; b Lishui Institute of Forestry, Lishui, China

**Keywords:** *Cerasus jingningensis*, chloroplast genome, phylogenetic analysis

## Abstract

*Cerasus jingningensis* is a deciduous shrub endemic to Zhejiang province, China. In our study, we assembled and characterised the chloroplast (CP) genome of *C. jingningensis* based on high-throughput Illumina sequencing data. The CP genome is 157,913 bp in length with an overall GC content of 36.7%, and it consists of two inverted repeats of 26,427 bp each, a large single copy of 85,934 bp, and a small single copy of 19,125 bp. The genome harbours 131 genes, including 85 protein-coding genes, 37 tRNA genes, eight rRNA genes, and a pseudogene ycf1. Phylogenetic analysis revealed that *C. jingningensis* is a sister of *C. serrulata var. spontanea*.

*Cerasus jingningensis* is a deciduous shrub with purplish brown bark, greyish brown branchlets, pink flowers and purplish black drupes, and it can be cultivated as an ornamental plant. *C. jingningensis* is a new plant species endemic to Zhejiang province, China, and it is found only in mountains of Jingning She Autonomous County and Longyou County, Zhejiang province. *C. jingningensis* is morphologically similar to *C. serrula* (Sect. Serrula), another *Cerasus* species which is distributed in provinces of Guizhou, Qinghai, Sichuan, Xizang, and Yunnan in China (Wu et al. 2003). *Cerasus* species belongs to Prunoideae of family Rosaceae, and in recent years, the complete chloroplast genome sequences of several Prunoideae plants have been sequenced and characterised, and these included *C. yedoensis*, *Amygdalus davidiana*, and *C. pseudocerasus* (Cho et al. 2016; Feng et al. 2018; Zhang et al. 2018). However, the complete CP genome of *C. jingningensis* has not been characterised, and its phylogenetic position is still unresolved. In our study, we reported the CP chloroplast genome of *C. jingningensis*.

Fresh leaves were collected at an altitude of 1478 m in Dayanghu mountain (27°52′16′′N, 119°44′22′′E), Jingning She Autonomous County, and total genomic DNA was extracted following the CTAB protocol (Doyle and Doyle 1987). A voucher specimen (CHS2018069) is stored at the Molecular Biology Laboratory in Taizhou University. A DNA library was constructed, and it was then sequenced using the Illumina Hiseq X Ten system (Illumina, CA, USA). Approximately 6 Gb raw data of 150 bp paired-end reads were obtained, and the clean reads were assembled by NOVOPlasty (Dierckxsens et al. 2017). The CP genome was annotated by Dual Organellar GenoMe Annotator (DOGMA), and the gene borders were corrected manually (Wyman et al. 2004). The CP genome *C. jingningensis* (GenBank accession MN175194) is 157,913 bp in length, consisting two inverted repeats (IR), a large single copy (LSC), and a small single copy (SSC), and the sequence lengths are 26,427 bp, 85,934 bp, and 19,125 bp, respectively. The overall GC content of the CP genome is 36.7%, while the GC percentages in IR, LSC, and SSC are 42.5%, 34.6% and 30.2%, respectively. The genome possesses 131 genes, including 85 protein-coding genes, 37 tRNA genes, eight rRNA genes, and a pseudogene. Among these genes, seven tRNAs (trnV-GAC, trnR-ACG, trnN-GUU, trnL-CAA, trnI-GAU, trnI-CAU, trnA-UGC), four rRNAs (rrn4.5, rrn5, rrn16, rrn23), and seven protein-coding genes (ycf2, ycf1, rps12, rps7, rpl23, rpl2, ndhB, and atpF) contain two copies, while one copy of ycf1 is a pseudegene.

Complete genome sequences of *A. kansuensis* (KF990036)*, A. davidiana* (NC_039735)*, C. pseudocerasus* (KX255667)*, C. cerasoides* (MF621234)*, Padus padus* (KP760072)*, P. serotina* (MF374324)*, Malus hupehensis* (NC_040170)*, A. mongolica* (KY073235)*, A. mira* (NC_040125)*, C. maximowiczii* (KP760071)*, C. takesimensis* (NC_039379)*, A. persica* (HQ336405)*, C. subhirtella var. subhirtella* (KP760074)*, C. yedoensis* (KU985054), and *C. serrulata var. spontanea* (KP760073) were downloaded from NCBI, and a phylogenetic tree was generated by PhyML 3.1 (Guindon et al. 2010). The results indicated that *C. jingningensis* grouped with *Cerasus* species, and it was closely related to *C. serrulata* var. *spontanea* (Sect. Serrula), a close relative of *C. serrula*, with a 100% bootstrap support (Figure 1).

**Figure 1.**
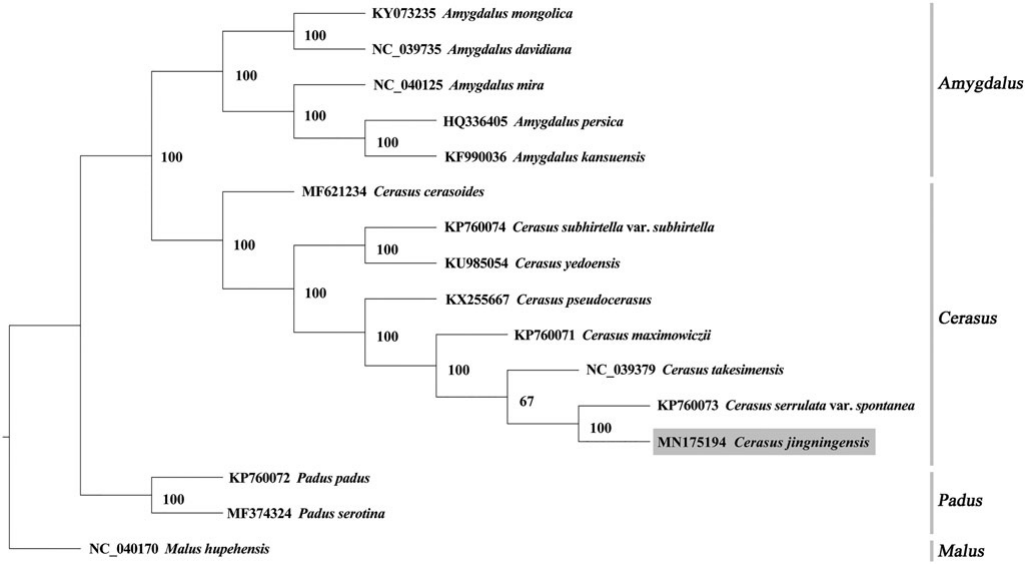
Maximum likelihood tree of 16 Prunoideae species based on chloroplast genome sequences, with *Malus hupehensis* as the outgroup. The numbers next to nodes are bootstrap support values.

## References

[CIT0001] Cho MS, Cho CH, Kim SY, Yoon HS, Kim SC 2016 Complete chloroplast genome of *Prunus yedoensis* Matsum.(Rosaceae), wild and endemic flowering cherry on Jeju Island, Korea. Mitochondrial DNA Part A. 27:3652–3654. 10.3109/19401736.2015.1079840 26329800

[CIT0002] Dierckxsens N, Mardulyn P, Smits G 2017 NOVOPlasty: de novo assembly of organelle genomes from whole genome data. Nucleic Acids Res. 45:e18 28204566 10.1093/nar/gkw955 PMC5389512

[CIT0003] Doyle JJ, Doyle JL 1987 A rapid DNA isolation procedure for small quantities of fresh leaf tissue. Phytochem Bull. 19:11–15.

[CIT0004] Feng Y, Liu T, Wang XY, Li BB, Liang CL, Cai YL 2018 Characterization of the complete chloroplast genome of the Chinese cherry *Prunus pseudocerasus* (Rosaceae). Conservation Genet Resour. 10:85–88.

[CIT0005] Guindon S, Dufayard JF, Lefort V, Anisimova M, Hordijk W, Gascuel O 2010 New algorithms and methods to estimate maximum-likelihood phylogenies: assessing the performance of PhyML 3.0. Syst Biol. 59:307–321. 20525638 10.1093/sysbio/syq010

[CIT0006] Wu ZY, Raven PH, Hong DY 2003 Flora of China. Vol. 9. Beijing: Science Press.

[CIT0007] Wyman SK, Jansen RK, Boore JL 2004 Automatic annotation of organellar genomes with DOGMA. Bioinformatics. 20:3252–3255. 15180927 10.1093/bioinformatics/bth352

[CIT0008] Zhang X, Yan J, Ling Q, Fan L, Zhang MR 2018 Complete chloroplast genome sequence of *Prunus davidiana* (Rosaceae). Mitochondrial DNA Part B. 3:888–889. 10.1080/23802359.2018.1501325 PMC7801015 33490544

